# AMD3100 Attenuates Post-Traumatic Osteoarthritis by Maintaining Transforming Growth Factor-β1-Induced Expression of Tissue Inhibitor of Metalloproteinase-3 *via* the Phosphatidylinositol 3-Kinase/Akt Pathway

**DOI:** 10.3389/fphar.2019.01554

**Published:** 2020-01-22

**Authors:** Weiwei Lu, Zhiyi He, Jia Shi, Zhenggang Wang, Wei Wu, Jian Liu, Hao Kang, Feng Li, Shuang Liang

**Affiliations:** Department of Orthopaedics, Tongji Hospital, Tongji Medical College, Huazhong University of Science and Technology, Wuhan, China

**Keywords:** AMD3100, CXCL12a/CXCR4, post-traumatic osteoarthritis, TIMP-3, PI3K/Akt signaling pathway

## Abstract

AMD3100 is a small-molecule inhibitor of the C-X-C motif chemokine ligand 12/C-X-C chemokine receptor type 4 (CXCL12/CXCR4) axis, while its role in aggrecan metabolism is unclear. We hypothesized that the AMD3100 modulates the transforming growth factor-β1 (TGF-β1)-induced expression of tissue inhibitor of metalloproteinase-3 (TIMP-3) in chondrocytes. We evaluated expression of CXCL12/CXCR4 and TIMP-3 in the knee joints of rats with and without osteoarthritis (OA) by immunohistochemistry, immunofluorescence, Western blotting, and enzyme-linked immunosorbent assay (ELISA). The rats were divided into sham control, destabilization of the medial meniscus/AMD3100-treated (DMM/AMD3100-treated), and DMM/phosphate-buffered saline (PBS)-treated groups. After 6 weeks, the rats were euthanized and subjected to histological and immunohistochemical analyses. Also, interleukin (IL)-1-pretreated primary chondrocytes were cultured in the presence of empty control (−, −), CXCL12a (+,−), CXCL12a + small interfering RNA (siRNA) CXCR4 (+,+), or CXCL12a + siNC (+NC), and the expression levels of target markers were evaluated by Western blotting and real-time reverse transcription PCR (RT-PCR). The CXCL12/CXCR4 levels were higher, and the expression of TIMP-3 was lower, in the OA rats compared to the healthy control rats. The rats in the DMM/AMD3100-treated group revealed a markedly decreased immunological response and mild pathology. Treatment with CXCL12a increased expression of aggrecan and disintegrin and metalloproteinase with thrombospondin motifs-5 (ADAMTS-5) and suppressed that of TIMP-3 in IL-1-pretreated primary chondrocytes. TGF-β1 increased expression of TIMP-3, and this increase was reversed by CXCL12a *via* the phosphatidylinositol 3-kinase (PI3K)/Akt signaling pathway. Moreover, these effects were inhibited by the CXCR4 antagonist AMD3100 and the PI3K inhibitor LY303511. In conclusion, inhibition of the CXCL12a/CXCR4 signaling axis maintained TIMP-3 expression *via* the PI3K/Akt pathway. Our findings provide insight into the mechanism by which AMD3100 prevents OA.

## Introduction

Osteoarthritis (OA) is a common chronic disease and a leading cause of chronic pain and physical disability, afflicting over 15% of the elderly population (Yelin et al., 2019). Unfortunately, there is no effective treatment for OA because its pathogenesis is unclear (Vinatier et al., 2016; Chen et al., 2017).

Progressive deterioration and loss of articular cartilage is crucial in the pathogenesis of OA (Onuora, 2015; Bortoluzzi et al., 2018). Type II collagen and aggrecan are important structural components of the cartilage extracellular matrix (ECM) and are the primary determinants of the mechanical properties of cartilage. Type II collagen has a three-helical network structure and provides the structural strength and elasticity of articular cartilage. Multiple aggrecan monomers bind to hyaluronan and to link proteins to fill the interstices of the collagen network, which plays a role in lubrication and resistance to compression. Because aggrecan prevents degradation of collagen fibrils, its loss is an important event in early stage OA (Wilusz et al., 2014; Carballo et al., 2017). Matrix metalloproteinases (MMPs), particularly the collagenase matrix metalloproteinase 13 (MMP-13), are involved in degradation of type II collagen (Wang et al., 2013a; Onitsuka et al., 2018), and disintegrin and metalloproteinase with thrombospondin motifs-5 (ADAMTS-5) is responsible for degradation of aggrecan (Malfait et al., 2010; Verma and Dalal, 2011; Larkin et al., 2015).

Tissue inhibitor of metalloproteinase-3 (TIMP-3) is an inhibitor of aggrecanases, which implies that TIMP-3 functions as an endogenous inhibitor of these enzymes (Kashiwagi et al., 2001; Li et al., 2014). Therefore, the mechanism by which TIMP-3 protein levels are regulated in patients with arthritic diseases warrants further investigation.

C-X-C motif chemokine ligand 12 (CXCL12), also known as stromal derived factor-1 (SDF-1), belongs to the CXC chemokine subfamily. In joint tissues, CXCL12 is produced mainly by synovial fibroblasts, while CXCR4, the G-protein coupled receptor, is expressed in chondrocytes (Kanbe et al., 2002; Nagasawa, 2014). CXCL12 plays a protective role at low concentrations, but can be destructive at high concentrations.(Kitaori et al., 2009; Villalvilla et al., 2014). CXCL12/C-X-C chemokine receptor type 4 (CXCR4) may play a crucial role in the progression of OA. CXCL12 levels are markedly increased in the knee-joint fluid of patients with rheumatoid arthritis and of those with OA. CXCL12 also regulates the catabolic activity of chondrocytes by stimulating the release of matrix metalloproteinases and aggrecanases *in vitro* (Kanbe et al., 2002; Chinni et al., 2006; Lu et al., 2016). The CXCL12/CXCR4 axis plays a major role in the repair of cartilage by acting as a chemoattractant for inflammatory and stem cells (Brand et al., 2005; Hu et al., 2013; Wang et al., 2017). The CXCL12/CXCR4 axis may therefore play dual roles in early stage OA. In this study, we evaluated the effect of the CXCL12/CXCR4 axis on TIMP-3 expression in rats with post-traumatic osteoarthritis (PTOA) and explored the underlying mechanism(s). First, we evaluated the levels of CXCL12/CXCR4 and TIMP-3 in rats with early stage OA compared to healthy control rats. Second, we induced OA in rats by destabilizing the medial meniscus (DMM) and assessed the effect of AMD3100 on progression of OA and expression of TIMP-3. Third, we extracted and cultured rat primary chondrocytes with untreated control, siNC + CXCL12a, CXCL12a, or small interfering RNA (siRNA) CXCR4 + CXCL12a and assayed the aggrecan (ACAN), transforming growth factor-β1 (TGF-β1), TIMP-3, and ADAMTS-4/5 protein and mRNA levels. Fourth, we explored the role of mitogen-activated protein kinase (MAPK) signaling in CXCL12/CXCR4-mediated activation of TIMP-3.

## Results

### Expression of TIMP-3 Was Low and That of the CXCL12/CXCR4 Axis Was High in Rats With OA

**Figure 1:**
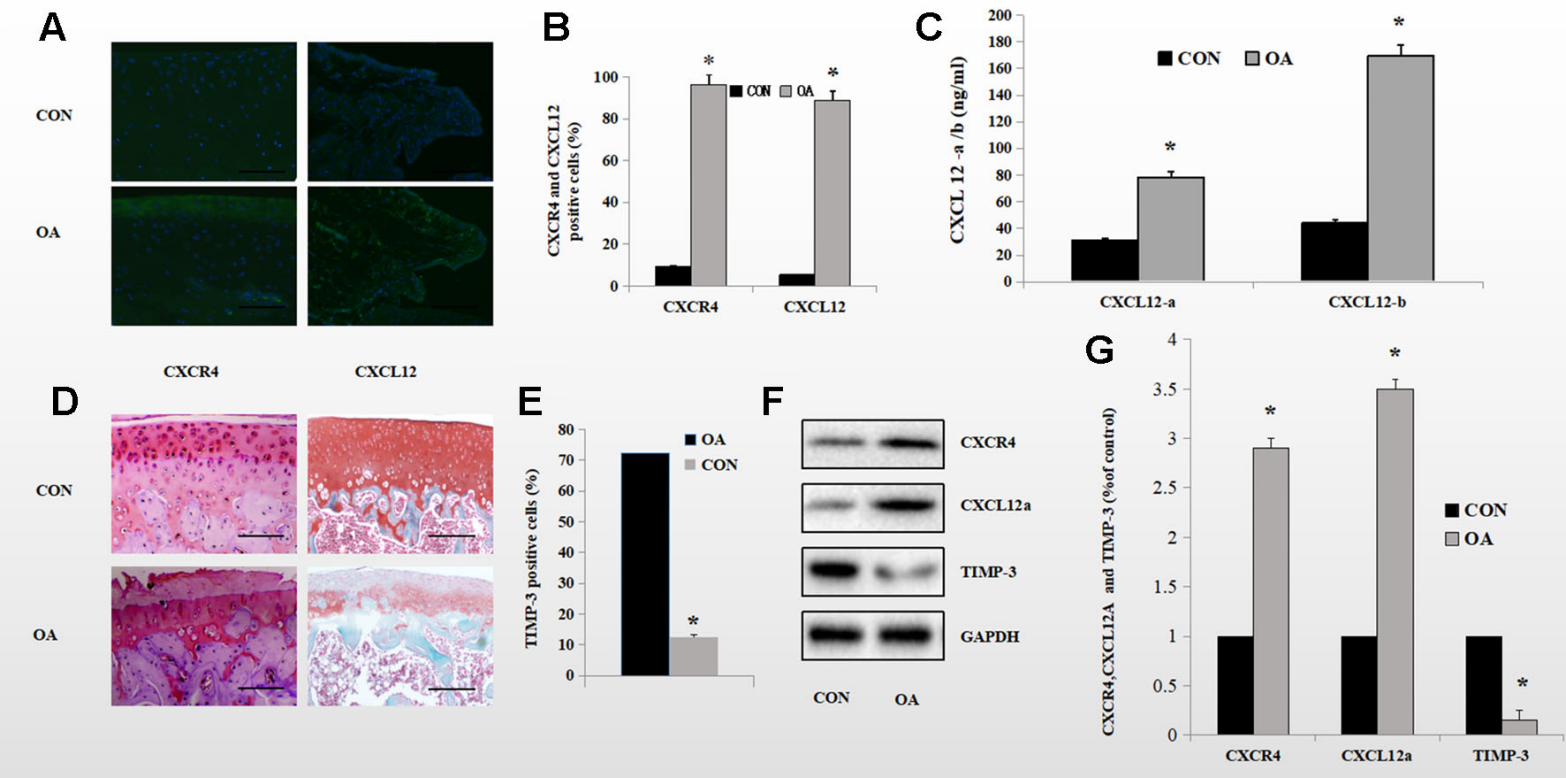
Expression of the CXCL12/CXCR4 axis and TIMP-3 in healthy control and OA rats. **(A, B)** Immunofluorescence analysis of CXCL12/CXCR4-stained synoviocytes and chondrocytes from healthy control and OA rats; quantitative data in **(B)** (n = 6 per group, *p < 0.05). **(C)** CXCL12a/b levels in the synovial fluid of healthy control and OA rats by ELISA (n = 5 per group, *p < 0.05). **(D)** TIMP-3 staining of superficial chondrocytes in the cartilage of healthy control and OA rats. Counterstaining by safranin orange; scale bar, 100 µm. **(E)** Number of TIMP-3-stained cells (n = 6 per group, *p < 0.05). **(F)** Western blotting of TIMP-3 and the CXCL12/CXCR4 axis in chondrocytes and synoviocytes from healthy control and OA rats; quantitative data in **(G)**. GAPDH served as the loading control (n = 6 per group, *p < 0.05).

We reported previously that SDF-1α induced expression of ADAMTS and speculated about the underlying mechanism. To investigate further the mechanism by which the CXCL12/CXCR4 axis mediates aggrecan metabolism, we determined the protein levels of components of the CXCL12/CXCR4 axis and of TIMP-3 in the knee synovium and cartilage of OA rats and healthy control rats using Western blotting. CXCL12/CXCR4-axis protein levels were significantly higher in OA rats than in healthy control rats. OA rats also exhibited lower TIMP-3 expression levels. (**Figures 1F, G**). Also, enzyme-linked immunosorbent assay (ELISA) revealed elevated CXCL12 protein levels in the knee synovial fluid of the OA rats (**Figure 1C**). Immunofluorescence staining showed that 92.2% of chondrocytes and 62.7% of synoviocytes in the OA rats were positive for CXCR4, compared to 11.2 and 5.2%, respectively, in the healthy control rats (**Figures 1A, B**). Furthermore, in the superficial zone of the cartilage of OA rats, 12.6% of chondrocytes were positive for TIMP-3 and there was considerable loss of proteoglycan (**Figures 1D, E**). These changes are directly related to aggrecan metabolism in OA. By comparison, 72.2% of chondrocytes were positive for TIMP-3 and the loss of proteoglycans was reduced in the healthy control rats (**Figures 1D, E**).

### AMD3100 Suppressed Cartilage Destruction and Alleviated the Severity of OA

**Figure 2:**
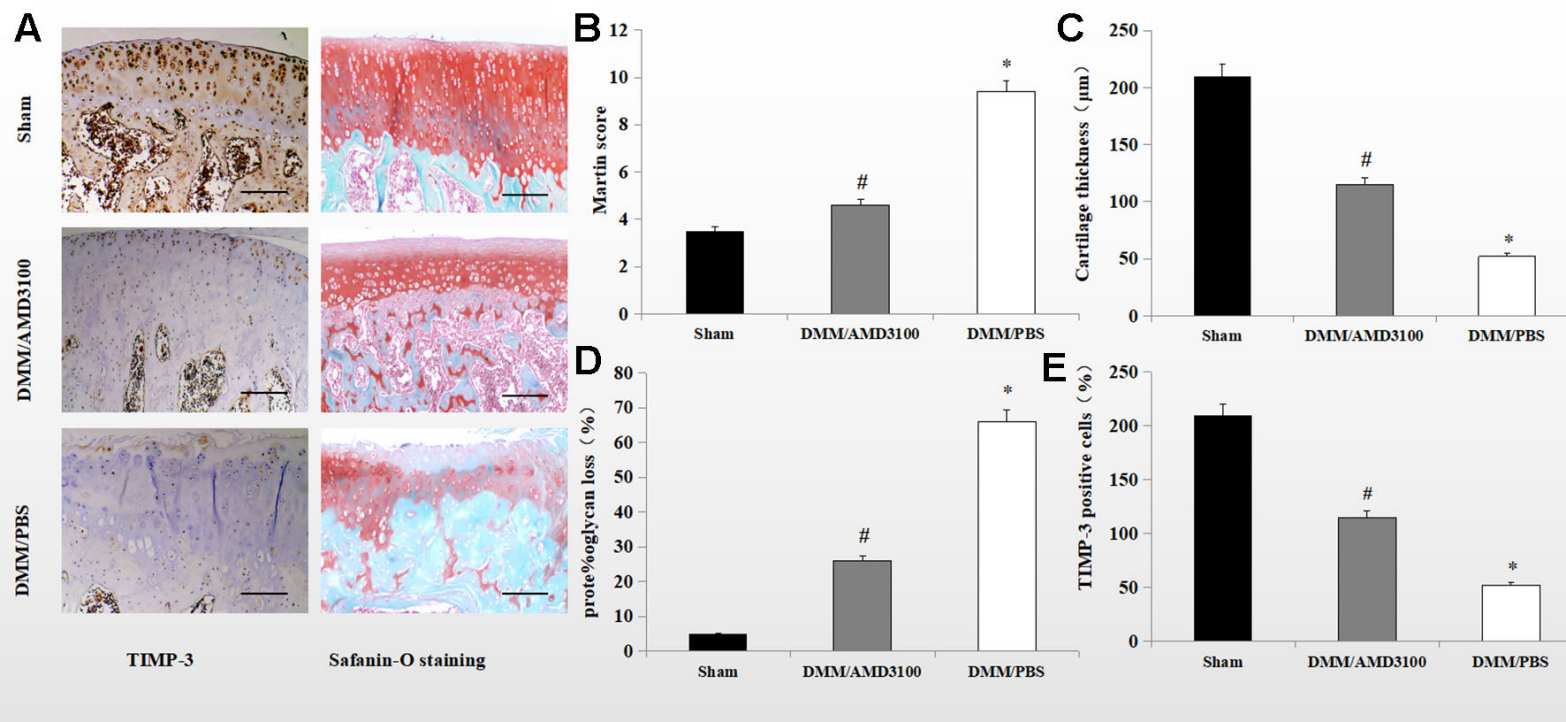
AMD3100 protects OA cartilage from proteoglycan loss and maintains TIMP-3 levels. **(A)** Safranin orange staining and immunostaining of TIMP-3 in knee sections from the sham, PBS-treated, and AMD3100-treated groups (scale bar, 100 µm). **(B)** Mankin scores of articular cartilage from the sham, AMD3100-treated and PBS-treated groups (n = 9 per group); **(C)** Cartilage thickness (n = 9 per group); **(D)** Proteoglycan loss from knee cartilage (n = 9 per group). **(E)** Number of TIMP-3 positive chondrocytes in knee cartilage (n = 9 per group). *DMM/PBS compared to sham group (p < 0.05); ^#^DMM/3100 compared to DMM/PBS group (p < 0.05).

Safranin orange staining and immunohistochemistry showed proteoglycan loss, cartilage damage, and decreased TIMP-3 expression in the knee joints of DMM/phosphate-buffered saline (PBS)-treated rats. Cartilage destruction was significantly alleviated in the sham control- and DMM/AMD3100-treated groups (**Figure 2A**). The Mankin score was lower (4.6 points; p < 0.05) in the DMM/AMD3100-treated group than in the DMM/PBS-treated group (9.4 points); while the scores of the sham control- and DMM/AMD3100-treated groups did not differ significantly (p > 0.05; **Figure 2B**). The rats in the DMM/PBS-treated group also showed significantly attenuated cartilage thickness (**Figure 2C**) and a significantly reduced level of proteoglycan (**Figure 2D**). AMD3100 treatment prevented joint destruction and loss of proteoglycan, as indicated by the low Mankin score (**Figure 2B**), and preserved cartilage thickness (**Figure 2C**). Quantification of safranin orange staining of cartilage in the rats in the PBS-treated group revealed a 66.4% decrease in proteoglycan loss, while the rats in the sham control- and AMD3100-treated groups showed little proteoglycan loss (**Figure 2D**). Finally, TIMP-3 expression was maintained by AMD3100 (p < 0.05; **Figure 2E**).

### Blocking the CXCL12/CXCR4 Axis Maintained the Interleukin-1-Induced Expression of TIMP-3 and Aggrecan, and Enhanced the Expression of ADAMTS-5 and TGF-B1

**Figure 3:**
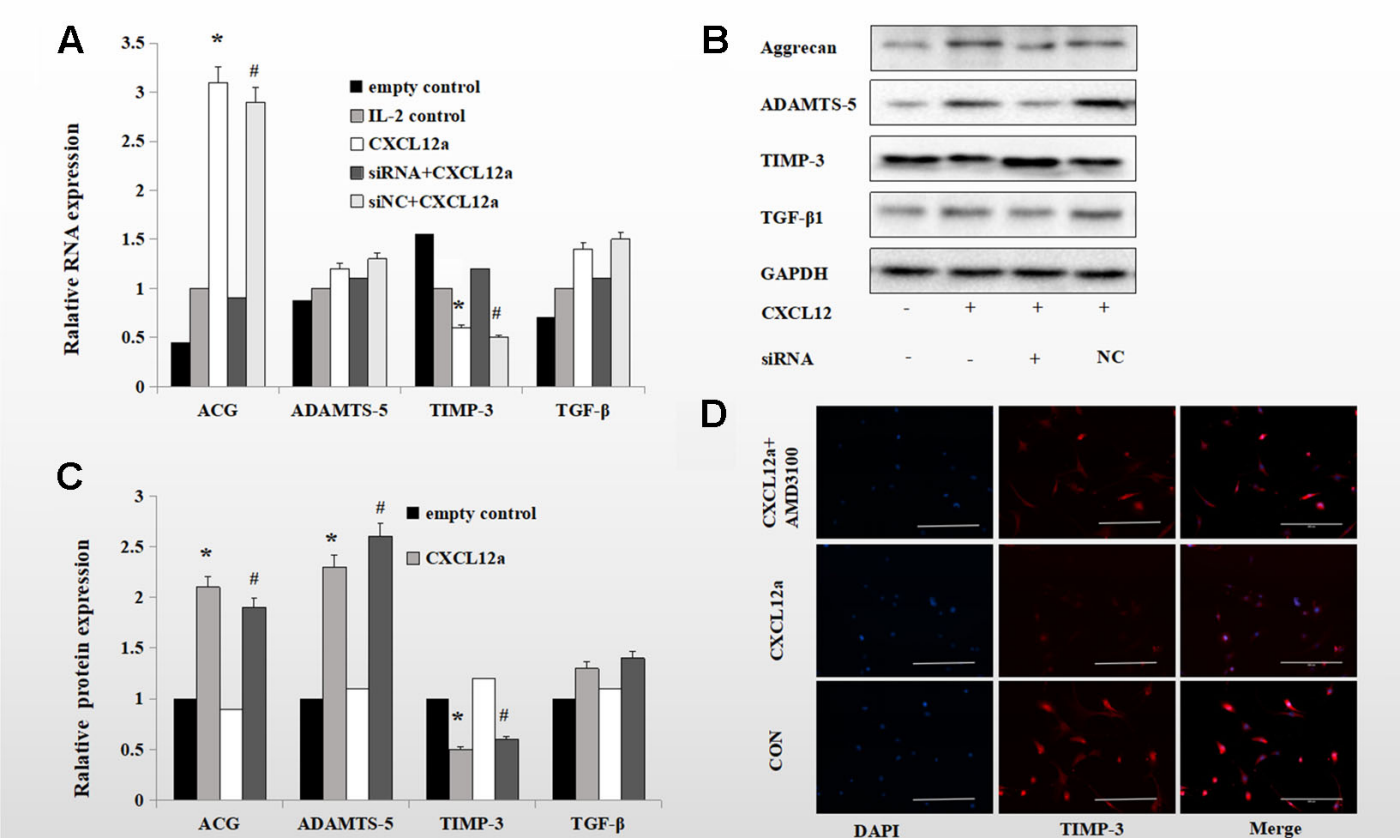
Blocking the CXCLL2/CXCR4 axis maintained IL-1-induced expression of TIMP-3 and aggrecan and enhanced expression of ADAMTS-5 and TGF-β1. **(B)** Primary chondrocytes were cultured in the presence of empty control, IL-2 control, CXCL12a (+,−), CXCL12a +siRNA CXCR4 (+,+) or CXCL12a + siNC (+NC) for 72 h and subjected to Western blotting of the protein levels of aggrecan, ADAMTS-5, TIMP-3, and TGF-β1. GAPDH served as the loading control (n = 4 per group). **(C)** Densitometric analysis. **(A)** Real-time PCR of aggrecan, ADAMTS-5, TIMP-3, and TGF-β1 mRNA levels in IL-1-induced primary cartilage treated with empty control (−,−), CXCL12a (+,−), CXCL12a +siRNA CXCR4 (+,+), or CXCL12a + siNC (+NC) for 24 h; *CXCL12a compared to empty control (p < 0.05); ^#^siNC + CXCL12a compared to CXCR4 + CXCL12a siRNA (p < 0.05). **(D)** Primary chondrocytes were cultured in the presence of empty control, CXCL12a (250 ng/ml) or CXCL12a + AMD3100(20 μM) for 48 h. Immunofluorescence staining for TIMP-3; representative images are shown. TIMP-3 was visualized using a goat anti-rabbit IgG, and nuclei were stained blue by DAPI. Scale bar, 200 µm.

To verify the role of the CXCLL2/CXCR4 axis in TIMP-3 activation *in vitro*, we assessed the effect of CXCL12a on markers of aggrecan metabolism—aggrecan, ADAMTS-5, TIMP-3, and TGF-β1. Rat primary chondrocytes pretreated with or without interleukin (IL)-1 (24 h, 10 ng/ml) were cultured with empty control, IL-2 control, CXCL12a, siRNA CXCR4 + CXCL12a, or siNC + CXCL12a *in vitro* for 24 h, and the aggrecan, ADAMTS-5, TIMP-3, and TGF-β1 mRNA and protein levels were assayed. Treatment with 250 ng/ml CXCL12a significantly increased aggrecan and ADAMTS-5 mRNA levels (p < 0.05) but did not significantly affect those of TGF-β1 or ADAMTS-5. Chondrocytes treated with the CXCR4 siRNA exhibited similar TGF-β1 and ADAMTS-5 mRNA levels to the control (**Figure 3A**). Primary chondrocytes cultured with 250 ng/ml CXCL12a for 72 h exhibited markedly increased expression of aggrecan and ADAMTS-5 (p < 0.05) and significantly decreased expression of TIMP-3, but TGF-β1 protein levels were unaffected (**Figure 3B**, p < 0.05). Primary chondrocytes pretreated with IL-1 and CXCL12a (250 ng/ml) for 72 h exhibited weak TIMP-3 staining (**Figure 3C**). Therefore, CXCL12a suppressed TIMP-3 expression.

### AMD3100 Maintained Expression of TIMP-3 and Smad3

**Figure 4:**
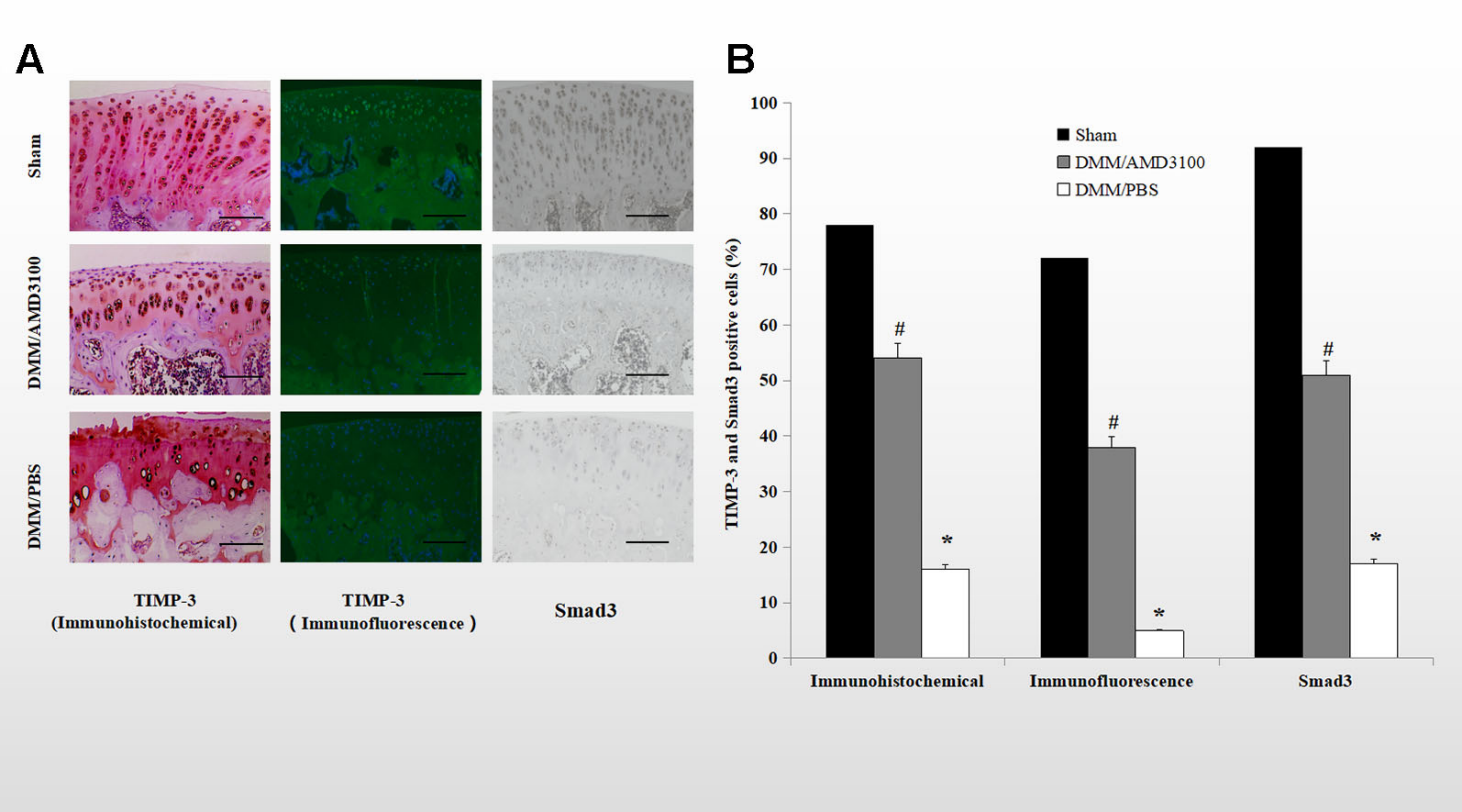
Blocking the CXCLL2/CXCR4 axis maintained TIMP-3 and Smad3 expression. **(A)** Cartilage sections from the sham, DMM/AMD3100-treated, and DMM/PBS-treated groups were subjected to immunohistochemical and immunofluorescence analysis of TIMP-3 and Smad3. Scale bar, 100 µm. **(E)** Numbers of cells positive for TIMP-3 and Smad3 (n = 9 per group, p < 0.05). *DMM/PBS compared to sham (p < 0.05); ^#^DMM/AMD3100 compared to DMM/PBS (p < 0.05).

Immunohistochemical and immunofluorescence analyses showed that both TIMP-3 and mothers against decapentaplegic homolog 3 (Smad3) were expressed in cartilage of the rats in the sham group. The percentages of TIMP-3 and Smad3 positive cells were significantly lower in the cartilage of the rats in the DMM/PBS-treated group (16.0 and 5.4% of all chondrocytes, respectively), while AMD3100 prevented the loss of TIMP-3 and Smad3 in the OA rats (54.4 and 38.3% of all chondrocytes, respectively) (**Figures 4A, B**; p < 0.05). Blocking the CXCLL2/CXCR4 axis maintained the expression of Smad3; the AMD3100-treated group showed significantly more stained cells compared to the PBS-treated group (51.9 and 17.7% of all chondrocytes, respectively) (**Figures 4A, B**; p < 0.05).

### AMD3100 Maintained TIMP-3 Expression Mediated by TGF-β1 by Inducing Akt Phosphorylation

**Figure 5:**
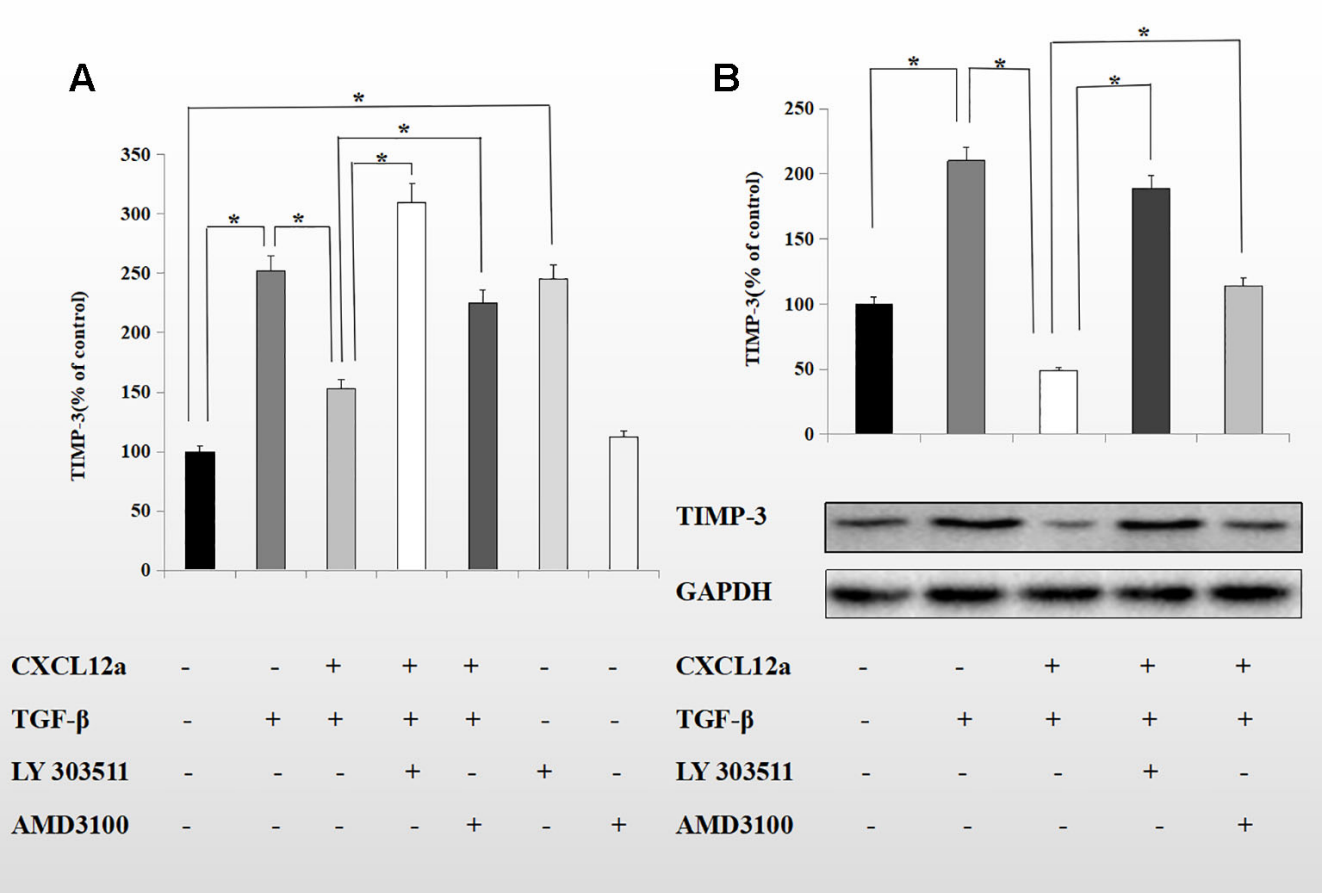
CXCL12a suppressed TGF-*β*1-induced elevated expression of TIMP-3 *via* the Akt pathway. Primary chondrocytes were treated with TGF-*β*1 (10 ng/ml) for 1 h, followed by LY303511 (10 *μ*M) or AMD3100 (20 *μ*M) for 1 h and CXCL12a (250 ng/ml) for 72 h. **(A)** TIMP-3 mRNA level by real-time RT-PCR. **(B)** TIMP-3 protein level by Western blotting. Differences between treatments were analyzed using one-factor ANOVA and were considered significant for p < 0.05. (n = 4 per group, *p < 0.05).

CXCL12a mediates Akt phosphorylation, and Akt regulates TGF-β1 signaling by directly interacting with Smad3 (Conery et al., 2004). To probe the mechanism underlying the inhibitory effect of CXCL12a on TGF-β1-induced TIMP-3 expression, primary chondrocytes were treated with TGF-β1 (10 ng/ml) for 1 h, followed by LY303511 (10 μM) or AMD3100 (20 μM) for 1 h, and finally SDF-1α for 72 h. The mRNA and protein levels of TIMP-3 were analyzed using real-time PCR and Western blotting (**Figures 5A, B**). CXCL12a inhibited the TGF-β1-induced increase in TIMP-3 mRNA levels; this effect was blocked by LY303511 (TGF-β1 + CXCL12α *vs*. TGF-β1 + CXCL12α + LY303511, p < 0.05) and AMD3100 (TGF-β1 + CXCL12α *vs*. TGF-β1 + CXCL12α + AMD3100, p < 0.05). This result is consistent with our Western blot results. LY303511, an Akt inhibitor, prevented CXCL12α-mediated TIMP3 downregulation, suggesting that CXCL12a prevented the TGF-β1-induced increase in TIMP-3 expression by activating the phosphatidylinositol 3-kinase (PI3K)/Akt signaling pathway and inducing Akt phosphorylation.

**Figure 6:**
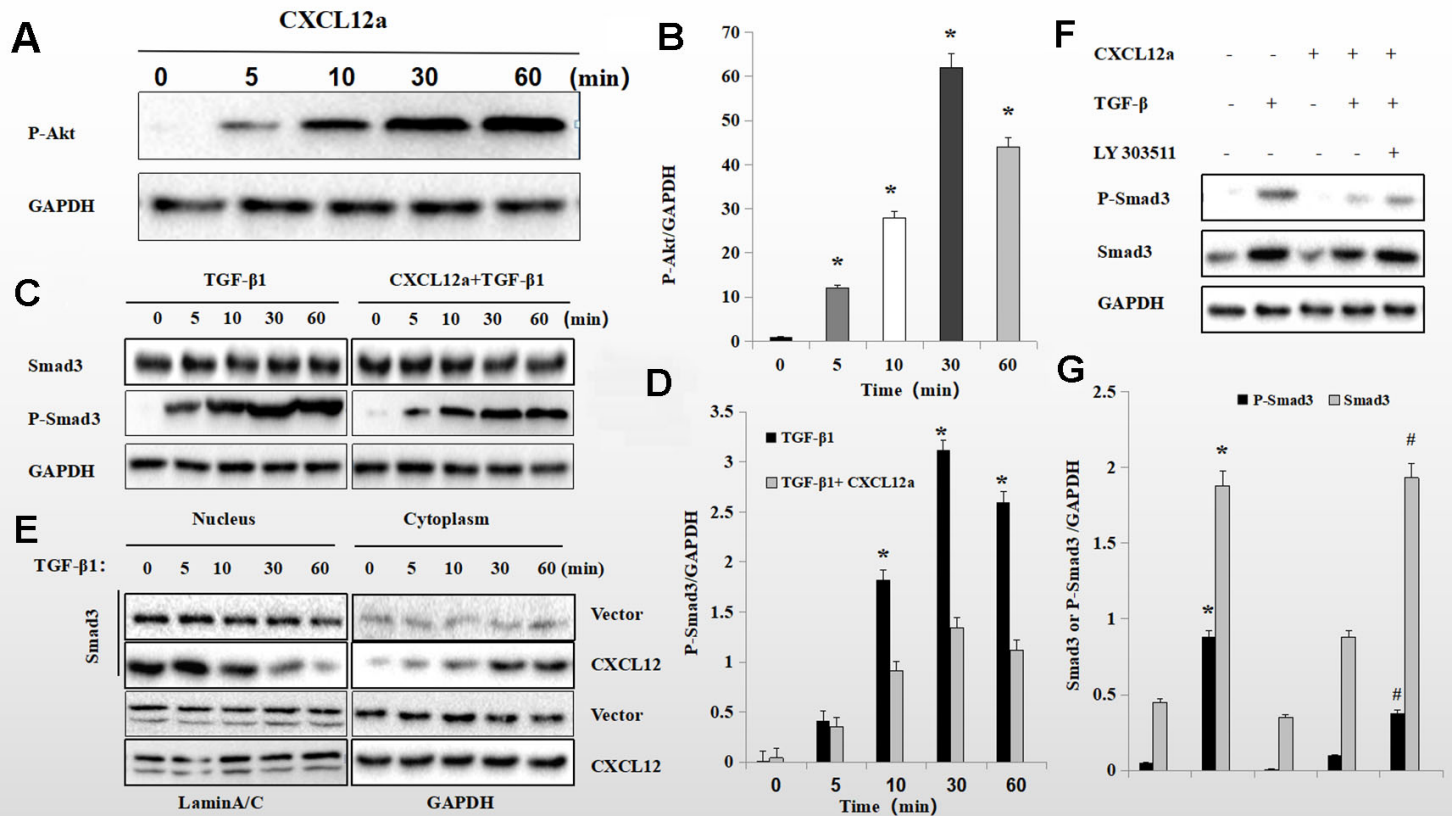
CXCL12a -induced phosphorylation of Akt downregulated TGF-β1-induced phosphorylation of Smad3. **(A C, E)** Rat primary chondrocytes were treated with CXCL12a (250 ng/ml), TGF-βp1 (10 ng/ml), or TGF-β1 (10 ng/ml) and CXCL12a (250 ng/ml) for the indicated times. Phosphorylated Akt, P-Smad3 and Smad3 levels by Western blotting. **(B, D)** Protein expression detected by western blotting was quantitated by densitometric analysis. (n = 4 per group, *p < 0.05). **(F)** Rat primary chondrocytes were treated with CXCL12a (250 ng/ml), TGF-β1(10 ng/ml), or Akt inhibitor (LY303511) for 48 h. P-Smad3 and Smad3 levels were determined by Western blotting. **(G)** Protein expression detected by western blotting was quantitated by densitometric analysis. Differences between treatments were analyzed using one-factor ANOVA and were considered significant for p < 0.05. *TGF-b1 compared to sham (p < 0.05); ^#^CXCL12a+TGF-b1+LY303511 compared to CXCL12a+TGF-b1 (n = 4 per group p < 0.05).

To assess further the interaction of the PI3K/Akt signaling pathway with the CXCL12a/CXCR4 axis and TGF-β1, primary chondrocytes were treated with CXCL12a, TGF-β1, or CXCL12a + TGF-β1. CXCL12a induced phosphorylation of Akt (**Figure 6**) and TGF-β1 induced phosphorylation of Smad3 (**Figure 6B**; p < 0.05). Also, application of both TGF-β1 and CXCL12a significantly suppressed TGF-β1-induced phosphorylation of Smad3 (**Figures 6C, D**; p < 0.05). Western blotting showed that TGF-β1 promoted phosphorylation of Smad3, which was suppressed by CXCL12a. CXCL12a acted upon the TGF-β1-Smad3 signaling pathway to exert biological effects. Total Smad3 expression in the cytoplasm and nucleus suggested that CXCL12t inhibited Smad3 nuclear translocation (**Figure 6**). To explore the function of the PI3K/Akt signaling pathway further, primary chondrocytes were treated with CXCL12a, TGF-β1, CXCL12a + TGF-β1, or an Akt inhibitor (LY303511) (**Figure 6**). The results showed that TGFb-induced phospho-Smad3 was maintained by Akt inhibitor treatment (**Figures 6F, G**).

## Discussion

Crucial risk factors may vary among joints and disease stages. It is difficult to distinguish between single and clustered risk factors associated with disease development or progression (Zhang et al., 2005). OA is a disease caused by many factors including gender, age, genetic factors, biomechanical changes, body mass index (BMI), nutritional factors, and bone mineral density (Pereira et al., 2015).

Loss of articular cartilage and subchondral bone sclerosis is a key characteristic of OA. Histologically, OA is characterized by early fragmentation of the cartilage surface, chondrocyte cloning, vertical clefts in the cartilage, variable deposition, remodeling, and violation of the tidemark by blood vessels (Pereira et al., 2015). Aggrecan is among the major structural components of cartilage, forming colossal aggregates with the glycosaminoglycan hyaluronan (HA), which is trapped within networks of collagen II. This composite structure endows cartilage extracellular matrix (ECM) with compressive strength and articular cartilage with shock-absorbing properties (Mead and Apte, 2018). Loss of aggrecan is a milestone in early stage OA, because aggrecan prevents loss of collagen fibrils (Scanzello et al., 2011; Verma and Dalal, 2011; Schmal et al., 2014; Apte, 2016). Therefore, guaranteeing the integrity of aggrecan is important for ameliorating OA. Under physiological conditions, aggrecan homeostasis is maintained by stable expression of aggrecanases and TIMP-3. If this homeostasis cannot be maintained, aggrecan is lost, leading to the development of OA (Huang and Wu, 2008; Lim et al., 2010). We reported previously that CXCL12/CXCR4 promotes expression of aggrecanase (Lu et al., 2016); however, why aggrecanase levels were related to neither loss of aggrecan nor the progression of OA was unclear.

We explored the correlation of CXCL12/CXCR4 and TIMP-3. First, we investigated expression of CXCL12/CXCR4 in OA and healthy control rats. Expression of the CXCL12/CXCR4 axis was increased and that of TIMP-3 was decreased in the OA rats, implying a link between the CXCL12/CXCR4 axis and TIMP-3-related cartilage loss (**Figure 1**). However, whether the CXCL12/CXCR4 axis controls activation of TIMP-3 in OA or is a causative agent of OA was unclear.

*In vivo*, TIMP-3 activation and cartilage loss were correlated with activation of the CXCL12/CXCR4 axis. Blockade of the CXCL12/CXCR4 axis maintained expression of TIMP-3 and alleviated cartilage loss by repressing the degradation of aggrecan, indicating a role in the progression of PTOA (**Figure 2**). The rats in the DMM/AMD3100-treated group showed less severe damage to cartilage, implying that AMD3100 alleviated PTOA-associated loss of articular cartilage (**Figure 2**). Also, stable expression of TIMP-3 was maintained by inhibiting expression of the CXCL12/CXCR4 axis (**Figure 2**). This is, to our knowledge, the first report of the function of the CXCL12/CXCR4 axis in TIMP-3-related cartilage loss.

There is a link between decreased TIMP-3 expression and ECM loss, and maintenance of TIMP-3 expression ameliorates OA-associated degradation of cartilage. *In vitro*, CXCL12a suppressed expression of TIMP-3 in primary chondrocytes (**Figure 3**). We found that CXCL12a activated aggrecanase in primary chondrocytes pretreated with IL-1 (**Figure 3**). Also, aggrecan levels were enhanced by CXCL12, indicating that the CXCL12/CXCR4 axis plays a dual role in aggrecan metabolism. ADAMTS-5 mRNA is a conserved sequence; effects on ADAMTS-5 appeared to be stronger at the protein level than at the transcriptional level, suggesting the involvement of post-transcriptional effects. When a stable state cannot be sustained, aggrecan is lost and the ECM is degraded. CXCL12a had little effect on TGF-β1 mRNA and protein levels in IL-1-treated primary chondrocytes. CXCL12a increased ADAMTS-5 protein levels but had little effect on mRNA levels, implying posttranscriptional regulation.

TIMP-3 may have potential for inhibiting cartilage loss (Dunn et al., 2014; Li et al., 2014). Unlike the other members of the TIMP family, TIMP-3 is insoluble and remains tightly bound to the matrix (Brew et al., 2000). *In vivo*, TIMP-3 is regulated by the TGF-β1 signaling pathway. TGF-β1-induced expression of TIMPs is a key factor in the progression of OA and is regulated by Smad proteins (Cross et al., 2005; Leivonen et al., 2013; Wang et al., 2013b; Zhu et al., 2017). Smad2/3 signaling mediates TGF-β1-induced expression of TIMP-3, and the gene encoding TIMP-3 is a target of Smad signaling (Qureshi et al., 2008). In the absence of Smad3, mice are deficient in aggrecan and type II collagen due to activation of MMP13 *via* p38 and Runx2(Chen et al., 2012). As a component of the intracellular TGF-β1 signaling pathway, Smad3 promotes expression of TIMP-3, which plays a critical role in aggrecan homoeostasis (Di Sabatino et al., 2009; Medina et al., 2011; Leivonen et al., 2013). During the development of OA, CXCL12 regulates numerous homeostatic and pathological processes *via* its receptor CXCR4 by activating several signal transduction pathways, including the PI3K/Akt pathway (Xue et al., 2017; Lin et al., 2018). It is, therefore, possible that cross-talk between the Smad and the PI3K/Akt pathways influences ECM production.

CXCL12a suppressed TGF-β1-induced elevated expression of TIMP-3. The CXCL12-related signaling pathway includes PI3K/Akt, MAPK, nuclear factor-kappaB, and Wnt/β-catenin (Corr, 2008; Tian et al., 2013; Xue et al., 2017; Yin et al., 2019). Activation of the PI3K/Akt pathway influences the adhesion, survival, migration, and proliferation of cells (Yano et al., 2007). In this study, CXCL12 inhibited expression of Smad3 and of its target gene, TIMP-3. Therefore, SDF-1α likely regulates expression of TIMP-3 *via* Smad3. We found that AMD3100 reversed the TGF-β1-induced downregulation of TIMP-3 expression in primary chondrocytes. These effects of CXCL12 were reversed by LY303511, implying that the PI3K/Akt pathway was involved in the process of CXCL12-induced decrease in TIMP-3 expression. Thus, activation of the PI3K/Akt pathway interferes with Smad3 phosphorylation, preventing TGF-β1-induced TIMP-3 expression. Moreover, CXCL12a-induced Akt phosphorylation impeded TGF-β1-induced Smad3 phosphorylation and the PI3K inhibitor LY303511 blocked the CXCL12a-mediated suppression of TGF-β1-induced expression of TIMP-3. Therefore, CXCL12a modulates TGF-β1 signal transduction by promoting Akt phosphorylation, suppressing Smad3 expression, and reducing TIMP-3 production in primary chondrocytes.

Taken together, the data imply that PTOA-associated aggrecan loss can be relieved by blocking the CXCL12/CXCR4 axis, which activates the PI3K/Akt pathway and suppresses the TGF-β1-induced Smad pathway. This enhances our understanding of the role of the CXCL12/CXCR4 axis in the development of PTOA. However, the detailed mechanism of CXCR4 signaling for ECM remodeling requires further study.

## Methods

### Reagents and Antibodies

AMD3100 (ab120718, Purity: > 99%), LY303511 (ab145193) and primary antibodies for CXCL12 (ab25117), CXCR4 (ab124824), aggrecan (ab36861) ADAMTS-5 (ab41037), Smad3(ab52903), TIMP-3 (ab39184) and TGF-β1 (ab92486) were purchased from Abcam (Cambridge, UK), p-Akt (ab38449), p-Smad3 (ab122028) were purchased from Optomics (Hermiston, OR, USA).CXCL12a and CXCL12b ELISA kits were purchased from Tsz Biosciences (Boston, MA, USA). Alzet osmotic mini-pumps (2006) were purchased from DURECT Corporation (Cupertino, CA, USA). Recombinant rat CXCL12a, IL-1 and other reagents were purchased from Beyotime (Shanghai, China).

### Animals and Experimental Design

Sprague-Dawley rats were purchased from the Experimental Animal Center of Tongji Medical College (Wuhan, China) and were raised at the Animal Care Facility of Tongji Medical College as described previously. Animal studies were authorized by the Institutional Animal Research Committee of Tongji Medical College, and all experimental protocols involving animals were approved by the Institutional Animal Care and Use Committee (number TY20130286; 14 December 2013). Thirty 8-week-old male Sprague-Dawley rats (200 g ± 10 g) were divided randomly into three groups. The rats in the DMM/AMD3100-treated (n = 10) group were anesthetized with pentobarbital sodium, underwent destabilization of the medial meniscus (DMM) of the right knee as described by Glasson et al. (Glasson et al., 2007), and were infused with AMD3100 (3 mg/day) using an osmotic mini-pump (model 2006). The rats in the DMM/PBS-treated group (n = 9) underwent the same surgical procedure on the right knee as those in the DMM/AMD3100-treated group but were infused with phosphate-buffered saline (PBS) using an osmotic mini-pump. The rats in the sham control group (n = 11) underwent sham surgery on the right knee and did not receive infusion. All of the rats were euthanized at 6 weeks after surgery.

### Protein Extraction and Western Blotting

Whole protein was extracted from cell lysates and quantified using a Bicinchoninic Acid (BCA) Protein Assay Kit (Thermo Scientific, Rockford, IL, USA). Nuclear and cytoplasmic proteins were extracted using a Protein Assay Kit purchased from Beyotime (P0028; Shanghai, China).Total cellular extract (20 µg per well) was loaded on 8–15% (according to molecular weight) sodium dodecyl sulphate-polyacrylamide gels and transferred to polyvinylidene difluoride membranes (Millipore, Boston, MA, USA) by electroblotting. The membranes were blocked with 5% skim milk at room temperature for 1 h, and incubated with the appropriate primary antibodies overnight at 4°C. The membranes were then washed and incubated with the corresponding secondary antibodies at room temperature for 1 h. Finally, immunoreactive protein bands were visualized by chemiluminescence (Boster, Wuhan, China) using the ChemiDoc™ XRS+ System with Image Lab™ software (Bio-Rad, Hercules, CA, USA). The membranes were re-probed with a monoclonal anti-glyceraldehyde 3-phosphate dehydrogenase (GAPDH) antibody (Cell Signaling Technology, Boston, MA, USA) as a loading control.

### Cell Culture and Treatment

Primary rat chondrocytes were obtained from the knee cartilage of new-born Sprague-Dawley rats as described previously. The knee cartilage of new-born rats was cut into pieces in bioclean PBS. The knee cartilage sections were treated in trypsin-ethylenediaminetetraacetic acid (EDTA) for 20 min at 37°C and digested with collagenase (Beyotime, Shanghai, China) in complete Dulbecco's modified Eagle's medium (DMEM) for 2 h. Next, chondrocytes were percolated and cultured in 2:3 DMEM:F12 containing 10% fetal bovine serum (FBS) and 0.25% L-glutamine. Because primary chondrocytes undergo dedifferentiation during repeated passages, 0-generation chondrocytes were used in all experiments (Gosset et al., 2008).

### Real-Time Reverse Transcription-Polymerase Chain Reaction Analysis

Quantitative real-time reverse transcription-PCR (qRT-PCR) was conducted as described previously (Lu et al., 2016). In brief, chondrocytes were digested in TRIzol reagent (Invitrogen, Grand Island, NY, USA) and total RNA was extracted utilizing an RNeasy Mini Kit (Invitrogen, Carlsbad, CA, USA) according to the manufacturer's instructions. First-strand cDNA was synthesized using Moloney murine leukemia virus (MMLV) reverse transcriptase (Promega, Madison, WI, USA). For real-time PCR, cDNA was amplified using SYBR Green Master Mix (Invitrogen). All procedures were performed at least three times, and gene expression levels were normalized to that of *GAPDH*. The following primer pairs were used: forward, 5′-CTTCCCAACTATCCAGCCAT-3′; reverse, 5′-CTGCTGTAAAGGTTGACGGTGTA-3′ for rat aggrecan; forward, 5′-CTCCATGCAGCTTTCACTGT-3′; reverse, 5′-TCAGAATTTGGAATCGTCGTG-3′ for rat ADAMTS-5; forward, 5′-TATAGCAACAATTCCTGGCGTTAC-3′; reverse, 5′-TGTATTCCGTCTCCTTGGTCA-3′ for rat TGF-β1; forward, 5'GTGGTGGGAAAGAAGCTGGTGAA-3′; reverse, 5′-TGGCAAGATTAGTGTCCGGG-3′ for rat TIMP-3; forward, 5′-GGCACAGTCAAGGCTGAGAATG-3′; reverse, 5′- GGTGGTGAAGACGCCAGTA -3′ for rat GAPDH.

### Enzyme-Linked Immunosorbent Assay

Synovial fluid was obtained from the knees of healthy control rats and PTOA rats. Concentrations of CXCL12a and CXCL12b in the synovial fluid were determined using the corresponding enzyme-linked immunosorbent assay (ELISA) kits (Tsz Biosciences, Boston, MA, USA), according to the manufacturer's instructions.

### RNA Interference Assay

RNA Interference was performed as described previously. The small interfering RNA (siRNA) against CXCR4 was obtained from Transheep Bio (Shanghai, China). Gene silencing was conducted according to the manufacturer's instructions. Briefly, primary rat chondrocytes were treated and incubated for 72 h. When the cell density reached ~50%, 5 µl of Lipofectamine 2000™ (Invitrogen) was diluted in 250 ml of Opti-MEM medium and incubated for 5 min at room temperature. The CXCR4 siRNA and empty siRNA were diluted in 250 µl of Opti-MEM medium and treated with Lipofectamine 2000™. Next, the samples were incubated for 20 min at room temperature. The siRNA-lipid mixture was added to the samples, which were incubated at 37°C in a 5% CO_2_ atmosphere for 6 h. Finally, Opti-MEM was replaced with 2:3 DMEM:F12 containing 10% FBS and 0.25% L-glutamine for 24 h. The sequences of the CXCR4 siRNAs were as follows: 5t-GCGAGGUGGACAUUCAUCUTT-3t (sense), 5t-UUCUCCGAACGUGUCACGUTT-3t (antisense).

### Histological Assessment

At the time of euthanasia, the knee joints were isolated and fixed in 10% formalin for 48 h, decalcified in 10% EDTA (pH 7.4) for 14 days, and embedded in paraffin. Next, sagittal-oriented sections of the knee joints were stained with safranin orange and hematoxylin and the severity of OA was assessed using the modified Mankin scoring system (Mankin et al., 1971; Ostergaard et al., 1999). All of the samples were evaluated by three blinded independent observers, and the mean scores of each joint were calculated.

### Immunohistochemistry and Immunofluorescence

Immunohistochemistry was conducted utilizing a standard protocol. Sections of rat cartilage were analyzed using the DakoREAL™ EnVision™ Detection System (Dako, Glostrup, Demark), followed by counterstaining with 0.02% safranin orange. For immunofluorescence, chondrocytes or sections were fixed and incubated with the indicated primary antibodies, followed by the corresponding fluorophore-conjugated secondary antibodies (Invitrogen). Nuclei were stained with 4',6-diamidino-2-phenylindole (DAPI). The slides were incubated at room temperature in darkness for 1 h. Fluorescence intensity was quantitated using Image Pro Plus software (Media Cybernetics, Rockville, MD, USA). The numbers of positively stained cells in the whole cartilage area of five sequential sections per rat were determined.

### Statistical Analysis

Experiments were performed at least three times with similar results. Data are means ± 95% confidence intervals. Student's *t*-test was used to compare differences between two groups; and a multifactorial analysis of variance (ANOVA) for comparisons of three or more groups. The level of significance was defined as p < 0.05. The data were analyzed using Statistical Package for the Social Sciences (SPSS) 15.0 software.

## Data Availability Statement

The raw data supporting the conclusions of this article will be made available by the authors, without undue reservation, to any qualified researcher.

## Ethics Statement

The animal studies were authorized by the Institutional Animal Research Committee of Tongji Medical College, and all experimental protocols involving animals were approved by the Institutional Animal Care and Use Committee (number TY20130286; 14 December 2013).

## Author Contributions

All authors listed have made substantial, direct, and intellectual contribution to the work and approved it for publication.

## Conflict of Interest

The authors declare that the research was conducted in the absence of any commercial or financial relationships that could be construed as a potential conflict of interest.
